# Single-port transoral robotic surgery for a rare tonsil mass

**DOI:** 10.1093/jscr/rjad197

**Published:** 2023-04-19

**Authors:** David Z Allen, Daniel Gorelik, Edward B Butler, Jun Zhang, Joshua J Kain

**Affiliations:** Department of Otorhinolaryngology—Head and Neck Surgery, McGovern Medical School at the University of Texas Health Science Center, Houston, TX, USA; Department of Otolaryngology —Head and Neck Surgery, Houston Methodist Hospital, Houston, TX, USA; Department of Radiation Oncology, Houston Methodist Hospital, Houston, TX, USA; Department of Hematology and Medical Oncology, Houston Methodist Hospital, Houston, Texas USA; Department of Otolaryngology —Head and Neck Surgery, Houston Methodist Hospital, Houston, TX, USA

## Abstract

Squamous cell carcinoma predominates as the most common malignant lesion of the oropharynx with human papilloma virus-associated disease now predominant over tobacco-related oropharynx cancer. Other rare malignant pathologies can manifest as visible neoplasms in these anatomic sites with varying degrees of symptoms such as dysphagia, odynophagia, otalgia, aspiration, hemorrhage, weight loss and dyspnea. We present a case of a rarely encountered primary oropharyngeal sarcoma managed by single-port transoral robotic resection and a selective cervical lymph node dissection followed by adjuvant radiotherapy.

## INTRODUCTION

Awareness of head and neck address neoplasms has vastly increased in the age of human papilloma virus (HPV)-related oropharyngeal squamous cell carcinoma (OPSCC). HPV-related OPSCC now accounts for the majority share of oropharynx malignancy as opposed to HPV negative OPSCC with an incidence of ~4.6 cases per 100 000 individuals in the United States [1]. This disease process affects a younger patient cohort with distinct risk factors when compared with traditional smoking and alcohol-related OPSCC [2]. This shift has led clinicians to be much more attuned to mucosal head and neck neoplasms in patients without the traditional high-risk behaviors of tobacco and alcohol use.

However, other malignant pathologies do manifest in the lymphatic-rich environment of the oropharynx including salivary carcinomas, lymphomas and sarcomas. Clinicians must be vigilant to recognize less commonly encountered malignant pathologies in these anatomic sites. This surgical case report describes a rare primary tonsillar malignancy managed by primary transoral robotic surgery (TORS).

## CASE REPORT

A 37-year-old Latino male presented with a 1-month history of an enlarging, painless right tonsillar mass noticed during routine oral self-care. He self-referred to our tertiary care academic otolaryngology-head and neck surgery clinic with a clearly visible right tonsillar 1 cm violaceous exophytic mass arising from the tonsil tissue into the oropharyngeal lumen. An in-office transoral biopsy was performed.

His social history was notable for active vaporized nicotine use and prior polysubstance abuse. He identifies as a homosexual male in a monogamous relationship with multiple prior sexual partners. He started pre-exposure prophylaxis (PREP) for human immunodeficiency virus (HIV) with a combination of pharmacologic emtricitabine/tenofovir 2 months prior to the onset of the lesion. As part of initiating this PREP regimen, he received screening testing which was negative for HIV by serology and polymerase chain reaction (PCR) testing.

**Figure 1:**
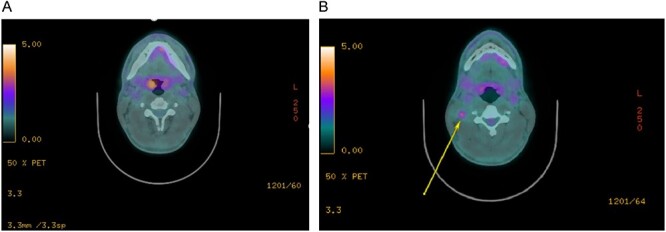
Staging imaging. (**A**) PET-CT imaging of the right tonsil primary lesion. (**B**) PET-CT imaging of the right level IIb avid node.

Staging imaging by positron emission tomography-computed tomography (PET-CT) showed right tonsil avidity and a suspicious level IIb ipsilateral node (Fig. 1A and B). Repeat labs were obtained with high-sensitivity PCR and serologic testing for HIV and CD4 cell counts. This testing did not reveal any interval HIV seroconversions and normal lymphocyte counts. His medications and medical history were carefully examined, and infectious disease specialists were engaged, though no immunocompromising conditions were identified. Importantly, no skin lesions or distant disease was identified on whole-body PET-CT.

The initial in-office transoral biopsy specimen returned as Kaposi’s sarcoma with immunohistochemical positivity for CD31, CD34, Vimentin and Human Herpes Virus 8. The case was discussed in our multi-disciplinary head and neck oncology tumor board, which recommended locoregional control by transoral robotic resection and selective neck dissection, given his HIV negativity and lack of immunocompromising risk factors.

He declined an elective neck dissection despite the avid ipsilateral level II node out of concern for the transcervical incision necessary to perform the lymphadenectomy portion of the procedure. We counseled the patient that in addition to gaining accurate staging and even therapeutic lymph node resection, the transcervical approach allows for ligation of the external carotid arterial system to mitigate postoperative catastrophic hemorrhage risks [3, 4]. Ultimately, he opted for an ultrasound-guided fine needle aspiration of the PET avid node at the time of transoral resection, with plans for a staged neck dissection if this yielded a positive or indeterminate result.

He underwent transoral robotic-assisted right radical tonsillectomy by a single-port technique (da Vinci Surgical System, Intuitive Surgical, Sunnyvale, CA, USA) in conjunction with an ultrasound-guided needle biopsy of the right level II suspicious node. He tolerated the procedure well and was discharged home once demonstrating an adequate oral intake and achieving oral pain control.

He presented to the hospital emergency department on postoperative Day 2 with an episode of small volume hemoptysis while at home. He was hemostatic and hemodynamically stable on arrival. His nodal fine needle aspirate also returned that day, showing suspicion for malignancy and identifying spindle cells. The patient was counseled and readily consented to a selective therapeutic neck dissection and external carotid supply ligation to address his nodal disease burden and hemorrhage risk. He was taken to the operating room that evening and underwent an ipsilateral right selective neck dissection of levels II–IV, as well as ligation of the ipsilateral linguofacial trunk of the external carotid system.

**Figure 2:**
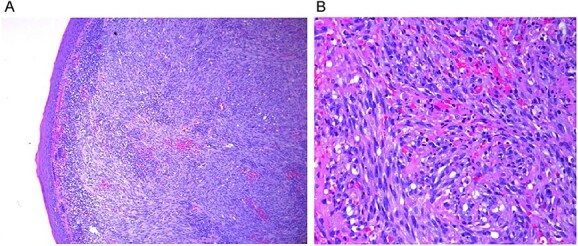
Histopathology of the primary lesions. (**A**) Low power view. (**B**) High power view.

His final pathology revealed a 1.2 cm right tonsil Kaposi’s sarcoma primary with clear margins but perivascular lymphoplasmacytic infiltration identified. The nodal dissection revealed Kaposi’s sarcoma in 3 of 42 lymph nodes examined. All nodal deposits were contained within the node and sub-centimeter in volume (Fig. 2A and B).

He was again presented in review at our institutional multidisciplinary head and neck oncology tumor board with recommendation for adjuvant radiotherapy for additive locoregional control. After completion of adjuvant therapy, he has been followed closely by the head and neck surgical oncology, radiation oncology and medical oncology teams according to the National Comprehensive Cancer Network recommended schedule for a minimum of 5 years post-treatment. He remains to be in a state of disease-free survival at the time of this article, 14 months post-treatment.

## DISCUSSION

Kaposi’s sarcoma is a disease entity typically associated with immunocompromised states, most notably of individuals with HIV/AIDS. Endemic forms of the disease are also well described and characterized in certain ethnic populations. Perhaps the least known at-risk population are HIV seronegative men having sex with men with limited cases reported to date [5–8]. Limited case-series data suggest that locoregional treatment in the HIV seronegative population offers encouraging rates of disease control [9, 10]. Our case here serves to add to the limited experience of the disease in this population and subsite. This case presentation demonstrates a rare presentation of an oropharyngeal mucosal Kaposi’s sarcoma in a male lacking traditional or immunocompromising or demographic risk factors treated by TORS, selective neck dissection and adjuvant radiotherapy.

## Data Availability

All data is available in the article and accompanying figures.
